# Keyword Index for Volume 91

**DOI:** 10.1038/sj.bjc.6602328

**Published:** 2004-12-21

14-3-3 *σ* 1131

15d-PGJ_2_ 633, 1074

[^18^F]FHPG 2079

[^18^F]FMISO-PET 1947

1p36 1119

3,3′-diindolylmethane 1358

4HPR 1821

5-fluorouracil 618, 1428, 1442, 1466

5-FU 1447

5-iodo-2′-deoxyuridine 366

667 COUMATE 1399

8-Cl-cAMP 186

9-*cis* retinoic acid 1561

*α*-interferon 1236

*α*-particle emitter ^213^Bi 2086

acute leukaemia 1160

acute lymphoblastic leukaemia 707

acyclovir 2079

adducts 1380

adenocarcinoma 739, 1384, 1853

adenocarcinoma of lung 771

adenocarcinoma of uterine cervix 1032

adenoid cystic carcinoma 1131

adenoma–carcinoma sequence 312

adenovirus 171, 1610

adhesion, Rho-GTPases 1800

adjuvant 1005

adjuvant chemotherapy 56

adjuvant therapy 242, 1656, 1893

adoptive immunotherapy 817

advanced 839

advanced breast cancers 861

advanced cancer 254, 1050

advanced gastric cancer 453

advanced NSCLC 1996

advanced soft tissue sarcoma 1639

age 1880

aggressive NHL 850

AKT 1420, 1735, 1808

aldehydes 111

allelic imbalance 1025

aluminium disulphonated phthalocyanine 788

American Joint Committee on Cancer stage IV 699

amputation 1012

androstenediol 1399

aneuploidy 558

angiogenesis 359, 498, 1149, 1174, 1293, 1391

angiogenesis inhibitor 1645

anthracycline 37

antibodies 1610

antibody engineering 1200

antibody processing 1500

antibody therapy 1500

antiinflammatory drug 525

antimelanoma activity 297

antioestrogen 1703

antisense 1166

antitumour activity 1624

anxiety 893

Apaf-1 1089

*APC* 1155, 1571

APE/ref-1 1166

*APITD1* 1119

apoptosis 164, 178, 195, 305, 359, 537, 580, 589, 803, 1195, 1358, 1372, 1405, 1614, 1718, 1726, 1808, 1842, 1931, 2026, 2094

*ASC1* 985

aspirin 525

*ATM* 783

attendance 1887

audit 248

autoimmunity 1508

axillary clearance 651

*β*-catenin 153, 1571

Bacillus Calmette-Guérin 607

base excision repair 1166

Bax 1372, 1931

B-cell lymphoma 1500

Bcl-2 164, 171

Bcr-Abl 1735

behaviour therapy 1050

belief in curability 254

benign prostatic disease 1755

benzo[*a*]pyrene 1380

biliary tract cancer 1769

biological 530

biomarkers 129, 725, 1190

birth weight 519, 1666

bisphosphonates 803, 1703

bladder cancer 607, 844, 1025, 2034

BMS-184476 213

body mass index 519

bone marrow 1813

bone tumour 1858

borderline tumours 1829

brain 374

brain metastases 639, 829

brain tumour 1678

BRCA 1787

*BRCA1* 1155, 1910

BRCA1/2 1580

*BRCA2* 1155, 1910

breakage–fusion–bridge cycle 327

breast 237, 954

breast cancer 69, 96, 99, 178, 233, 242, 277, 305, 413, 476, 512, 519, 618, 639, 651, 868, 873, 879, 959, 1063, 1190, 1251, 1263, 1308, 1532, 1591, 1687, 1694, 1813, 1893

breast cancer cells 1703

breast cancer family history 884

breast cancer incidence 861

breast cancer mortality 861

breast cancer screening 861

breast density 233

breast neoplasms 644, 1482, 1666, 1795

breast radiography 1795

breast radiography, quality assurance 1795

C595 monoclonal antibody 2086

CA IX 1301, 1947

cachexia 408

calcitriol 1425

camptothecin 50

cancer 56, 111, 504, 907, 1269

cancer awareness 62

cancer cachexia 1742

cancer chemoprevention 1213

cancer genetics 1829

cancer registries 1663

cancer risk 1525

cancer screening 1795

cancer services 62

cancer survival 1663

cancer symptom management 822

cancer testis antigen 141

cancer-related fatigue 822

cancer-testis antigen 1566

capecitabine 613, 834, 839, 1019, 1763, 2005

carbohydrate antigen 15-3 873

carboplatin 213, 627, 683, 1038, 1651

carcinoembryonic antigen 873

carcinoembryonic antigen-related cell adhesion molecule 6 1384

carcinogenesis 119, 195, 633

carcinoma 1515

cardiotoxicity 45

case–control study 1829

caspases 1358, 1726

cathine 1726

cathinone 1726

caveolin 959

CCI-779 1420

CD10 1342

CD20 antigen 1500

CD4+ 541

CD8+ 541

CD95 164

*CDH13* 1139

CDKN2A 1112

cDNA microarray 1543

cell cycle 195, 803, 1842, 2026

cell cycle arrest 1364

cell proliferation 1181, 2042

central nervous system tumour 923

central venous access port devices 1045

centrosome 327

ceramide 1821

cerebral blood volume 374

cervical cancer 84, 1032, 2056

cervical neoplasia 942

cervical screening 942

cervical smear test result 1887

cervix 1269, 1902, 2063

cervix cancer 106

cervix neoplasms 530

cervix uteri 935

cetuximab 418

CGH 775, 2063

chemoprevention 381, 1364

chemoradiation therapy 1025

chemoradiotherapy 11, 673

chemoresistance 434

chemosensitivity 666

chemotherapy 18, 476, 695, 839, 844, 1005, 1038, 1239, 1425, 1428, 1614, 1639, 1758, 1763, 1769

childhood ALL 913

childhood cancer 37, 923, 1905

childhood leukaemia 916

children 425

chromosome 16 1591

chromosome 1p 1105

chromosome 3p 1143

chromosome 9q 760

chromosome aberrations 775

chromosome instability 327

chromosome rearrangements 753

cigarette smoke 111

cigarette smoking 1280, 1525

CIN1 2056

CIN3 942

circulating tumour cells 1813

cisplatin 18, 589, 683, 1614, 2005

classic Kaposi’s sarcoma 1261

clinical trial 1645

clodronate 1703

clonality 775

clusterin 1842

c-Myc 1372

cohort study 942

colon neoplasms 1718

colorectal cancer 153, 339, 344, 381, 839, 980, 1015, 1428, 1434, 1453, 1711, 1758, 1931

colorectal carcinoma 312

colorectal liver metastases 205

combination chemotherapy 1032, 1472

communication 254

comparative study 23

complementary therapies 1482

consultant volume 459

conversion 1269

cost of illness 77

cost–benefit analysis 23

cost-effectiveness 84

Cox regression 923

COX-2 633

CpG island 707

CPT-11 482, 1205, 1434, 1442, 1447

C-reactive protein (CRP) 205, 699, 1063, 1236, 1755, 1993

cross-sectional 1575

CSF 219

CT scan 850

CTCF 1591

CTL 398, 817

cultural competence 62

cumulative incidence 1229

cure models 225

CXCR1 1970

CXCR2 1970

cyclin D 1239

cyclin D1 153

cyclin E 1239

cyclo-oxygenase 1213

cyclooxygenase 1015

cyclooxygenase-2 359

cyclophosphamide 1466

CYFRA 21-1 873

CYP1A1 1380

CYP1B1 966, 1380

cystadenocarcinoma 2048

cytochrome *P*450 333

cytokeratin 18 739

cytokeratin 5 739

cytokeratin-19 1813

cytokeratin-19 fragments 873

cytokine 607

cytokine receptors 1993

cytology 558

cytoskeleton 1327

cytotoxic chemotherapy 1561

cytotoxic T lymphocyte 287

cytotoxicity 1195, 2026

DARPP-32 119

database 355

*DBCCR1* 760

DCIS 564

DDS 1775

DEC1 954

decision-making 855

dendritic cells 1656

depression 900

deprivation 1063

desmoplastic reaction 1316

development 258

DHA-paclitaxel 1651

diagnosis 714, 1633

diet 1624

dietetics 447

differential expression 1160

differentiation 258, 1143

dihydropyrimidine dehydrogenase 1245

direct IAP-binding protein with low pI 1349

*Dkk*-3 707

DNA 1066

DNA adducts 333

DNA damage checkpoint 1669

DNA hypermethylation 270

DNA methylation 703, 760

DNA replication licensing 262, 714

DNA vaccine 688

DNA-PKcs 1735

docetaxel 18, 618, 1425, 1466, 1996

doublet regimens 489

Down syndrome 1866

doxorubicin 1775

doxorubicin (adriamycin) 1800

drug resistance 1166, 1405, 1800

dysplasia 1081

early discharge 651

ECarboF 1472

economic burden 77

educational attainment 923

efficacy 1434

EGFR 418, 430, 1066, 1301, 2026, 2042

EGFR family 1532

EGFR-1 470

elderly 1453

elderly NSCLC patients 489

elongation factor-1 delta 282

endocytosis 270

endometrial cancer 720, 1808

endosonography 23

enterolactone 99

epidemiology 512, 525, 644, 935, 1261, 1275, 1829

epidermal growth factor receptor 208, 795

epidermal growth factor receptor tyrosine kinase inhibitor 208

epirubicin 18, 45, 1466

EPR effect 1775

Epstein–Barr virus 572

ER gene expression 1694

ER-*β* 1687

ErbB2 1200, 1916

ERK 589

erlotinib 418

ET-1 434

ET_A_R 434

ET_B_R 434

etoposide 659, 1038, 1842

Ets transcription factors 1308

evaluation 935

Ewing’s sarcoma 225, 1858

experimental therapeutics 1808

expression 1515

extracellular domain 430

extracellular matrix 1327

extracellular matrix proteins 1964

family cancer history 1575

family history 96, 929

Fas 164

Fas receptor 1718

Fas-associated phosphatase-1 1718

FBP 270

feasibility 627

FHIT 2056

Fhit deficiency 1669

Fhit protein 1669

first pregnancy 512

first-line chemotherapy 1466

FISH 558

flavonoids 1364

fludarabine 695

folinic acid 453

follicular 695

follicular thyroid tumours 732

fractionated cisplatin 844

fractionated irradiation 1543

fragile histidine triad gene (*FHIT*) 1571

fragile sites 753

free radicals 111

full length TRAIL 171

function 1858

functional analysis 1287

galectin-3 1096

ganciclovir 2079

gangliosides 389

gastric adenocarcinoma 11

gastric cancer 1139, 1245, 1924

gastric caner 1335

gastric carcinoma 18, 1342

gastrointestinal cancer 834

GD1b synthase 389

gefitinib 208, 418, 1964, 2026

gemcitabine 482, 489, 673, 844, 1166, 1763

gemcitabine ovarian cancer 627

Geminin 262

gene amplification 725

gene expression 666, 1015, 1089, 1597

gene polymorphism 1551

gene profiling 200

gene therapy 817, 1610, 2079

gene–environment interaction 339

genetic counselling 1580

genetic pathway 312

genetic risk 1575

Gleason score 1755

glioblastoma 1195

glioma 374, 745, 1174, 1656

glioma survival 1678

glycolysis 2094

granulocytes 1316

growth 519

growth factor receptor 666

growth inhibition 580

growth pattern 1293

growth regulation 1955

*GSTP1* 985

guidelines 4

Guthrie cards 913

haemoglobin oxygen saturation 788

haptoglobin 129

haptoglobin-1 precursor (HAP1) 129

Hashimoto’s thyroiditis 1096

HCC 1964

HD 850

head and neck cancer 1477

head and neck neoplasms 124

head and neck squamous cell carcinoma 2005

health anxiety 893

height 519

*Helicobacter pylori* 929

heparin 30

hepatoblastoma 972

hepatocarcinogenesis 1955

hepatocellular carcinoma 753, 1561

HER-2 430, 795

HER2/neu 959, 1195, 1308

HER3 2034

HER4 2034

Herceptin 1190

heregulins 2034

herpes simplex virus thymidine kinase 2079

herpes virus 913

herpes zoster 1275

hierarchical cluster analysis 319

hierarchical clustering 775

HIF-1*α* 954

high risk 69

high-dose 5-fluorouracil 453

high-grade gliomas 425, 1038

histocompatibility antigens class II 1500

histological subtype 1143

HLA-A24 287

HLH 1990

*hMLH1* 1155

*hMSH2* 1155

HNPCC 1155, 1287

*HO-1* 1551

Hodgkin’s disease 572

homeodomain 258

hormonal receptor 959

hormone replacement therapy 644

hospital teams 248

hospital volume 459

household and family 907

HPV 942, 1269

HPV16 2056

human 1880

human bronchial epithelial cells 333

human gastric mucin 1342

human melanoma cell lines 297

human papillomavirus 84

human telomerase reverse transcriptase 972

hypersensitive patients 1251

hypersensitivity 213

hypoalbuminaemia 205

hypopharynx 258

hypoxia 374, 954, 1181, 1947

I*κ*B 381

Id-1 2042

IFN-gamma 688

IGF1 1735

IGF-binding proteins 1525

IL-6 1074, 1335

IM862 1645

immune escape 1718

immune response 688

immune therapy 1880

immunohistochemistry 119, 434, 552, 1342, 1532, 1556, 1591

immunotherapy 398, 607, 688, 1200, 1656

inadequate 1887

incidence 77, 916, 1275

indicator 972

induction 1624

infections 1866

inflammatory cytokines 1993

information needs 855

information services/^*^standards/utilisation 1482

informative censoring 1229

inhibition of cell proliferation 2094

insulin-like growth factors 1525

integrins 1327

interaction 233

interferon 164

interferon-alpha 621

interleukin-6 1755

interleukin-8 1327

interleukin-8/CXCL8 1970

internet 1482

internet/^*^standards/utilisation 1482

interstitial therapy 441

interventional radiologists 1045

intestinal lesions 1571

intestinal polyps 1571

intratumoral CD8+ T cells 1711

invasion 745, 1308, 1384, 1556, 1800

iodine 544

ionising radiation 2026

IRES vector 1842

Iressa 208

irinotecan 659, 678, 1245, 1434, 1447, 1453

ischaemia reperfusion injury 788

JACC study 929

JAK 1074

Kaplan–Meier estimate 1229

Khat 1726

Ki-67 262, 2018

KIT 2048

knowledge 855

lactate dehydrogenase (LDH) 699

Laminin-5 1964

laser capture microdissection 1096

late cardiac toxicity 37

leptomeningeal metastasis 219

leucovorin 1442, 1758

leukaemia 186, 1866

leukaemia cells 1726

LightCycler 972

lignan 99

limb-sparing surgery 1012

LINE retrotransposons 985

liposomal doxorubicin 1639

liquid-based cytology 84

liver enzymes 205

liver perfusion 1610

Lokich 1447

longitudinal 907

long-term response 1873

loss of heterozygosity 760, 1105, 1143

loss to follow-up 106

lower extremity 1858

lower extremity sarcomas 1012

lung 1515

lung adenocarcinoma 1143

lung cancer 208, 537, 1149, 1280, 1970, 2018

lung carcinogenesis 1380

lymphangiogenesis 498

lymphatic metastasis 124

lymphoma 141, 695

MAG-CPT 50

MAGE 398

magnetic resonance imaging 23, 374

maintenance therapy 621

malignancy grade 644

malignant effusion 558

malignant melanoma 765

mammaglobin 1813

mammary carcinoma 1200

mammary gland 1372

mammography 413

mammography screening 242

MAPK 1405

mass screening 530, 900

maternal hypertension 1666

MCF-7 1821

MCF-7 cells 178

MCM 262

Mcm5 714

MDM2 1415

measles virus 572

medical oncology 900

MEK 589

melanoma 699, 803, 829, 1089, 1495

membrane transport 2079

menarche 519

mesoionic compounds 297

mesothelioma 9

Met expression 703

meta-analysis 2018

metabolism 1213

metabolites 333

metalloprotease inhibitor 30

metastases 1782

metastasis 359

metastatic 683

metastatic breast cancer 77, 1466

metastatic colorectal cancer 1447

metastatic lymph node 466

METH-2 1149

methotrexate 1428

methylation 707, 739, 771, 1131, 1149, 1335, 1835, 2071

methylselenol 195

MIC-A/B 1495

micelle 1775

microarrays 1112, 1160, 1614, 1924, 1931

MI-D 297

mismatch repair 1287

missing covariate data 4

mitogen-activated protein kinase 178, 795

mitomycin C 834, 839, 1624

mitomycin C resistance 1669

mitosis 537

mitoxantrone 1005

*MLL* cleavage and rearrangement 1990

MLPA 1155

MMP-9 1301, 1384

models 530

molecular markers 552

molecular staging 558

monitoring 873, 935

monochromatic synchrotron radiation 544

monoclonal antibody 725, 1195

mortality 242, 1902

motility 1800

mouth 1551

MSH6 1287

MTAP 1112

m-TOR 1420

MUC1 1633

MUC-1 mucin 2086

MUC2 1342

MUC4 1633

MUC6 1342

mucin 1633

mucoepidermoid carcinoma 1131

multicenter trial 45

multimedia 855

multimodality treatment 666

multiple breast carcinomas 775

multiple myeloma 1873

multiple testing 1160

multivariate analyses 1711

muscle 408

muscle wasting 1742

mutation 355, 2048

myoepithelial cells 564

natural product 1405

need for control 254

neoadjuvant chemotherapy 2012

neoplasm 1551

neoplasm seeding 9

nested case–control study 929

neuroblastoma 389, 1112, 1119, 1205, 1835

NF-*κ*B 381, 1742

NK 1495

NK911 1775

NKG2D 1495

NKT cells 1880

nonangiogenic growth 1293

non-small-cell lung cancer 430, 771, 1293, 1993

nonsteroidal anti-inflammatory drug 153

NQO1 1624

NSAIDs 381

NSCLC 171, 482, 1301

nuclear factor I-B3 1112

nuclear sites 916

nucleus 537

nude mice 1364

nurse led 651

nutrition support 447

oesophageal cancer 714, 1139, 1543, 1556

oesophageal neoplasms 282

oesophageal squamous cell carcinoma 119

oestrogen receptor 1703, 2012

oestrogen receptor beta 1694

oestrogens 178, 644, 1399

oestrone sulphate 1399

older people 907

oligoastrocytoma 1105

oligodendroglioma 262, 1105

oral 111, 1459

oral carcinoma 760

oral chemotherapy 613

oral contraceptives, ovarian cancer 1910

oral squamous cell carcinoma 1074

oral submucous fibrosis 1551

organotypic brain slice cultures 745

osteosarcoma 1858

outpatient 844

ovarian cancer 129, 621, 725, 1614, 1829, 1916, 2042

ovarian carcinoma 470

ovarian neoplasms 2048

ovarian tumour 633

overall survival probability 1229

oxaliplatin 453, 1428, 1758, 1931

oxygenation 1181

*p16*^*INK4a*^ 771

p21 262, 1239

P450 reductase 966

p53 178, 1025, 1131, 1415, 1614, 1931

p53 activity 1488

p53 gene mutation 1081

p53 overexpression 1081

p73 1025

paclitaxel 45, 489, 1639

paclitaxel (taxol) 1800

PAH 333

PAI-1 564

palliative 1005

palliative care 248, 900, 907, 1050

palliative radiotherapy 829

pancreatic cancer 459, 673, 1166, 1316, 1327, 1384, 1633

pancreatic cancer cell lines 2086

pancreatic tumours 2086

papillary carcinoma of the thyroid 703

papillomavirus 2063

parathyroid hormone-related protein (PTHrP) 287

PAS domain 141

pathological response 2012

pathology 23

patient survival 2034

pattern of failure 11

PAX8 732

PC3 cells 1358

PCR 913

PCR–SSCP 312

PDGFRA 2048

pegylated liposome 366

pentose cycle 2094

peptides 287, 1488

performance status 1236, 1453

perfusion 1181

peritoneal cytology 720

persistence 1269

PET scan 850

phaeochromocytoma 1835

pharmacogenetics 344, 678

pharmacokinetics 50, 1434, 1769, 1775

phase I 1459, 1651

phase I study 1032

phase I trial 1019

phase II study 1032, 1769

photodynamic therapy 441, 788

phyto-oestrogen 99

PI3K pathway 1420

pimonidazole 1947

place of death 907

platinum therapy 344

polygenic model 1580

polymeric 50

polymorphisms 344, 339, 783

population-based cohort study 923

population-based studies 1263

positron emission tomography 2079

postmenopausal 1666

PPAR*γ* 633, 732

predictive 1239

predictive factors 1263

predictive genetic testing 1787

predictive marker 1025

prenatal origin 913

preneoplasia 1515

preoperative 434

preoperative breast biopsy 1782

preoperative radiotherapy 1019

pretreatment parameters 1873

prevalence 1575

primary vaginal and cervical cancers 319

prodrug 366

progestin 644

prognosis 119, 237, 277, 430, 466, 470, 498, 552, 720, 829, 972, 1275, 1293, 1556

prognostic factors 96, 219, 282

prognostic index 829

prognostic marker 1391

prognostic models 4

prolactin 305

proliferative factor 2018

promoter methylation 1597

promoter polymorphism 765

pro-peptide 1955

prospective study 99

prostate 195, 1842

prostate cancer 287, 541, 580, 688, 855, 1005, 1358, 1425, 1472, 1755

prostate cancer susceptibility 783

prostate neoplasm 1853

prostate-specific antigen 688, 1853

prostatic intraepithelial neoplasia 739

proteasome expression 1742

protein degradation 408

protein expression 319

protein kinase A 186

protein synthesis 408

protein–protein interaction 1415

proteomics 129

proto-oncogene 282

psychological distress 504, 884

psycho-oncology 504

psychosocial 879

psychosocial aspects 504

psychosocial functioning 855

psychosocial interventions 1050

psychosocial issues 1787

PTEN 1420, 1808

*PTGS2/COX2* 339

purging 186

pyridoxal 5′-phosphate 1508

pyridoxal phosphatase 1508

Q-TWiST 1893

quality assurance 248

quality assurance, health care 1482

quality of life 56, 69, 1012, 1050, 1858, 1893, 1996

quality-adjusted survival 1893

quantitative real-time PCR 282

quantitative RT–PCR 1316

quercetin 1213

quinazoline 1066

radiation 868

radiation dose enhancement 544

radiation oncology 447

radiation therapy 1678

radical prostatectomy 1853

radioimmunoconjugate 2086

radioresistance 1543

radiosensitiser 366

radiosensitivity 666

radiosentitiser 673

radiotherapy 9, 23, 544, 868, 1239, 1251

Rapamycin 1420

RAR antagonists 580

*RARB2* 985

real-time RT-PCR 333, 1924

reassurance 893

receptor status 644

record linkage 92

rectal cancer 1019

rectal neoplasm staging 23

rectal neoplasms therapy 23

recurrence 2063

recurrence pattern 1342

recurrent head and neck cancer 441

redox metals 111

reflectance spectroscopy 788

registry 923

relative excess risk of death 1263

relative survival 1263, 1663

remission 893

renal cancer 1236

renal cell cancer 1645

renal cell carcinoma 164, 327, 966, 1349, 1763

reporting 4

resection 459

resistance 1205

response prediction 666

restaging 850

resveratrol 178, 333, 1380

retinoic acid 389

retinoic acid receptors 580

retroviral vector 1488

review 237

ribose synthesis 2094

rice bran 1364

risk 92, 1275

risk counselling 884

risk factors 45, 233

risk management 1787

risk modifiers 1580

ROC curve 1160

RT–PCR 200, 1813

S-1 1245, 1769

S100 739

S100A2 1515

safety 1434

saliva 111

salvage chemotherapy 659

sarcoma 237

satisfaction 884

satisfaction with information 1887

scaffold protein 1488

screening 69, 84, 413, 935, 1902

SDHB 1835

second line 659, 1996

second malignancy 868

second malignant neoplasm 1905

second mitochondria-derived activator of caspases 1349

second-line chemotherapy 482, 1442

selenium 195

selenomethionine 195

seminoma 683

sensitive relapse 659

sentinel lymph-node biopsy 124

sentinel node 1782

sequential paclitaxel 627

SEREX 141

serine protease 725

serology 1269

serous 2048

serum antibody 1566

serum lipids 476

serum marker 699

sex difference 929

SF-36 879

*SFRP1* 985

shedding 1500

siRNA 1384

skin cancer 1604

*SLIT1* 2071

*SLIT3* 2071

small GTPases 270

small molecule 1415

small-cell lung cancer 659

smoking 512

SN-38 678

socioeconomic status 879

*SOCS-1* 1335

solid tumours 1459

somatic 355

South Asian 62

southern Sardinia 1261

SPARC 1924

specialisation 459

sperm protein 17 1597

SRC-1 1687

stable isotope-based dynamic metabolic profiling (SIBDMAP) 2094

stage 720

stage IV breast cancer 77

standards 248, 1477

Stat3 1074, 1335

state anxiety 1887

statistical methods 1663

steroid sulphatase 1399

steroids 525

stomach cancer 929

stool 980

stromal macrophages 498

subcutaneous fibrosis 1251

sunlight 1604

surgery 23

survival 4, 106, 237, 459, 541, 720, 1236, 1275, 1453, 1477

survival analysis 1293

survivin 537

synergy 171

systematic review 1050, 2018

systemic inflammatory response 1063, 1236

TAA 398

Tabar classification 233

tamoxifen 476, 1251, 1694

targeted therapy 795

taxanes 213, 1005, 1651

TCC 398

telomerase 972

telomere 327

temozolomide 425

TFIID 1119

TGCT 589

TGF-*β* 1316

Tg-mRNA 200

thalidomide 1873

therapeutic intervention 966

therapy 1633

thymidylate synthase 1245

thyroglobulin 200

thyroid cancer 1096

thyroid carcinoma 200

tissue array 537

tissue inhibitor of matrix metalloproteinase 3 (TIMP-3) 1556

tissue microarray 1089, 1532

T-lymphocytes 541

topoisomerase I inhibitor 1459

topoisomerase II*α* 1038

total PSA 1755

toxicity 1453

*TP53* 1119, 1916

transcription factor 1566

transforming growth factor *α* 1955

transgenic mice 1372

transitional cell carcinoma 552

transudate 558

trastuzumab 639, 959

treatment choice 56

treatment-related leukaemia 1990

trends 1902

triazene 1066

troglitazone 1561

tumorigenesis 1372, 1566

tumour growth 1964

tumour immunity 1711

tumour M2-PK 980

tumour marker 699

tumour necrosis factor 30

tumour prestages 1955

tumour progression 1349, 1694

tumour screening 980

tumour size 277, 959

tumour suppressor gene 753, 1105, 1571

tumour vaccination 1656

tumour volume 205

tumours 237, 374, 954, 966, 1947, 2071

tyrosine kinase 1735

tyrosine kinase inhibitor 1174, 1391

Tyrphostin AG1024 1735

UFT 613, 1758

UGT1A1 678

ulcerative colitis 1081

underexpression 732

uPAR 564

urban and rural area 1261

urokinase plasminogen activator (uPA) 1308

uterine cervical cancer 466

utility 1893

UVC resistance 1669

uvea 1495

vaccine 817

vascularity 1181

VDR 765

VEGF 219, 498, 1174, 1181, 1391

VEGF-C 466

VEGFR2 1391

venous thromboembolism 92

vinorelbine 489

vitamin D 1425

waiting times 1477

website 355

weekly schedule 1996

worry 1787

WWOX gene expression 753

xenograft 1205

X-linked inhibitor of apoptosis 1349

XR11576 1459

XRCC1 1604

ZD1839 208

ZD6474 1174

zinc-finger protein 1566

